# Development of a PRISMA extension for systematic reviews of health economic evaluations (PRISMA-EconEval): a project protocol

**DOI:** 10.1186/s13643-025-02911-2

**Published:** 2025-11-11

**Authors:** Phuong Bich Tran, Joseph Kwon, Andrew Booth, Anastasios Bastounis, Ewan M. Tomeny, Sophie Staniszewska, Richard Grant, Kednapa Thavorn, Dalia Dawoud, Rafael Pinedo-Villanueva, Sally Hopewell, Matthew J. Page, Stavros Petrou

**Affiliations:** 1 Nuffield Department of Primary Care Health Sciences, University of Oxford, Oxford, UK; 2 Sheffield Centre for Health and Related Research (SCHARR), University of Sheffield, Sheffield, UK; 3 Department of Clinical Sciences, Liverpool School of Tropical Medicine, Liverpool, UK; 4 Warwick Research in Nursing, Warwick Applied Health, Warwick Medical School, University of Warwick, Coventry, UK; 5 Patient and Public Involvement (PPI) Representative, PPI Advisor to Universities of: Warwick, Oxford, Liverpool, and Coventry City Council, Coventry, UK; 6 Ottawa Hospital Research Institute, The Ottawa Hospital, School of Epidemiology and Public Health, University of Ottawa, Ottawa, Canada; 7 Science Policy and Research Programme, National Institute for Health and Care Excellence (NICE), London, UK; 8 Clinical Pharmacy Department, Faculty of Pharmacy, Cairo University, Giza, Egypt; 9 Nuffield Department of Orthopaedics, Rheumatology and Musculoskeletal Sciences, University of Oxford, Oxford, United Kingdom; 10 NIHR Oxford Biomedical Research Centre, Oxford, UK; 11 Methods in Evidence Synthesis Unit, School of Public Health and Preventive Medicine, Monash University, Monash, Australia

**Keywords:** PRISMA, Reporting guideline, Systematic review, Health economic evaluation, Consensus building, Delphi

## Abstract

**Background:**

Systematic reviews of health economic evaluations are key for evidence-based decisions but lack standardised reporting. This project aims to develop a Preferred Reporting Items for Systematic reviews and Meta-Analyses (PRISMA) extension for systematic reviews of health economic evaluations (PRISMA-EconEval).

**Methods:**

Project stages include the following: (1) scoping review, (2) Delphi surveys, (3) consensus meeting, (4) piloting, and (5) finalisation and dissemination. The project is overseen by the international multidisciplinary PRISMA-EconEval Management Group (PMG), Advisory Group, and Patient and Public Involvement Group.

1. The scoping review aims to identify candidate reporting items, with the protocol published elsewhere. The global applicability of these items to systematic reviews of health economic evaluations will be evaluated using sample papers from the scoping review, supplemented by nominations from the health economics community or other sources, where necessary.
2. A multi-round online Delphi survey will be conducted to achieve consensus on items for inclusion. A purposive sample of panellists (approximately 200) will be selected, ensuring representation of the following: health economists, systematic reviewers, information specialists, guideline developers, journal editors, healthcare decision-makers, research funders, and public representatives. Across two to three rounds, panellists will use a 1–9 scale to rate each candidate item’s ability to represent the minimum required for reporting, be relevant to all systematic reviews of health economic evaluations, facilitate complete and transparent reporting, and support the quality assessment of both the review and included studies.
3. An online consensus meeting (approximately 30 participants) will refine the wording of items and resolve any disagreements by vote.
4. Health economists independent of the project will apply the draft guidelines to a sample of published studies and identify practical challenges.
5. The PMG will meet to finalise the wording and presentation of the reporting items, ensure consistency with PRISMA 2020, and produce an explanation and elaboration document. Dissemination channels will include peer-reviewed health economics journals, conferences, and the EQUATOR network.

**Discussion:**

PRISMA-EconEval aims to improve clarity, consistency, transparency, quality, and overall value of systematic reviews of health economic evaluations. This will benefit researchers, peer reviewers, editors, decision-makers, and ultimately patients and the public through supporting decisions on healthcare resource allocation.

## Background

A systematic review summarises the available research evidence and, when done well, should provide an unbiased, reliable synthesis of the current state of knowledge on a topic of relevance to evidence-based decision-making [1]. The Preferred Reporting Items for Systematic reviews and Meta-Analyses (PRISMA) 2020 statement was designed to enhance the reporting of systematic reviews and meta-analyses — primarily of the effects of interventions, though it is also applicable to systematic reviews of studies with different objectives — by improving their clarity, consistency, transparency, quality, and overall value [2].

Health economic evaluations compare two or more healthcare interventions in terms of their costs and consequences [3]. Systematic reviews of health economic evaluations differ from other types of systematic reviews in their search strategy, study selection, data extraction, assessment of reporting and methodological quality of included studies, approaches to synthesis, and assessment of relevance and transferability of the outcomes [4]. Further complexity arises from the different contexts of health economic evaluation, including evaluations conducted alongside clinical studies with patient-level data and evaluations using decision modelling, with considerable methodological heterogeneity across these categories.

Health technology assessment, pricing, and reimbursement authorities in many countries require systematic reviews of health economic evaluations of varying methodological rigour to inform their decision-making [5]. The purpose of these reviews would depend on the type of end-user, but may include the following: (i) summarising existing knowledge for decision-makers and researchers; (ii) justifying and contextualising the modelling and analytical approaches in submissions considered by agencies and informing the development of new decision models, where appropriate; (iii) assessing whether published evidence is sufficiently reliable and generalisable to a local context, rendering further analysis unnecessary; and (iv) reducing error and bias in the abstraction and adjustment of results. The aim is to minimise opportunity costs and prevent suboptimal resource allocation from decisions based on incomplete or misleading evidence. Furthermore, well-reported systematic reviews of health economic evaluations could potentially play a crucial role in empowering patients and the public to make informed decisions, understand healthcare value, and participate in shaping healthcare policy.

A literature search of PubMed Central of studies published between 1st January 2015 and 25th March 2017 found 202 systematic reviews of health economic evaluations listed within this 27-month period [6]. Extending the search to other databases and grey literature and incorporating studies published after this time period are likely to increase this number substantially. The absence of reporting guidelines specifically for systematic reviews of health economic evaluations increases the risk that the research community and other stakeholders who rely on this type of research evidence will make inappropriate/costly decisions due to poor or incomplete reporting, with concomitant negative consequences for population health and wellbeing.

The introduction of reporting guidelines has progressively improved reporting quality across applied health research over time [7]. Numerous extensions to the widely adopted PRISMA reporting guidelines have been developed, including preferred reporting items for literature searches [8], scoping reviews [9], reviews of harms [10], reviews incorporating network meta-analyses [11], equity-focussed reviews, reviews of diagnostic test accuracy [12, 13], reviews of acupuncture interventions [14], and systematic reviews and meta-analyses of studies with individual participant data [15]. However, the PRISMA guidelines have not yet been extended to cover systematic reviews of health economic evaluations, highlighting the gap for specific reporting guidelines in this area. The ‘Development of a PRISMA extension for systematic reviews of health economic evaluations (PRISMA-EconEval)’ project addresses this gap. The development of standards for reporting will enhance completeness, transparency, and structure in the reporting of systematic reviews of health economic evaluations and generate user-friendly tools for authors, editors, peer-reviewers, and stakeholders that facilitate better reporting.

PRISMA-EconEval reporting items will have generic applicability to systematic reviews regardless of the type of health economic evaluation (i.e. cost-minimisation analysis, cost-benefit analysis, cost-effectiveness analysis, cost-utility analysis, cost-consequences analysis) and vehicle for health economic evaluation (e.g. economic evaluations based on randomised controlled trials, economic evaluations based on observational studies with patient-level data, economic evaluations based on decision-analytic modelling) adopted by the individual studies they cover. Health technology assessment agencies differ in their preferred methodological approaches to health economic evaluation, and these are further subject to change over time. We will therefore aim to generate a list of reporting items with international reach across jurisdictions and preferred methodological approaches which are likely to remain useful for years to come, but will be updated as needed [16, 17].

Moreover, the reporting items will be agnostic to debates on the utility of single study-based versus decision-modelling-based economic evaluations, recognising both the diversity of methodological approaches employed by analysts and the continued reliance of the research community and end-users on evidence from different study types [17]. Furthermore, although variation in health care practices, relative prices of resource inputs, and the adopted health benefit measures have tended to limit the potential for statistical pooling of cost-effectiveness estimates, the synthesis methods items within the initial list of reporting items will not preclude reference to methods such as meta-analysis and meta-regression. Recent methodological developments indicate advances towards standards for meta-analysis of a range of cost-effectiveness outcomes [18, 19]. We will, therefore, aim to future-proof the PRISMA-EconEval reporting guidelines with an inclusive approach to the application of synthesis methods. In summary, our focus is on promoting the reporting quality of systematic reviews of health economic evaluations, rather than their methodological quality, though it is likely that the reporting guidelines will indirectly enhance the methodological rigour of these systematic reviews.

## Objective

The objective of this study is to develop an extension to PRISMA for the reporting of systematic reviews of health economic evaluations (PRISMA-EconEval).

## Methods

This project is funded by the National Institute for Health and Care Research (NIHR) Research for Patient Benefit Programme (grant number NIHR206833) and hosted at the Nuffield Department of Primary Care Health Sciences, University of Oxford.

**Fig. 1:**
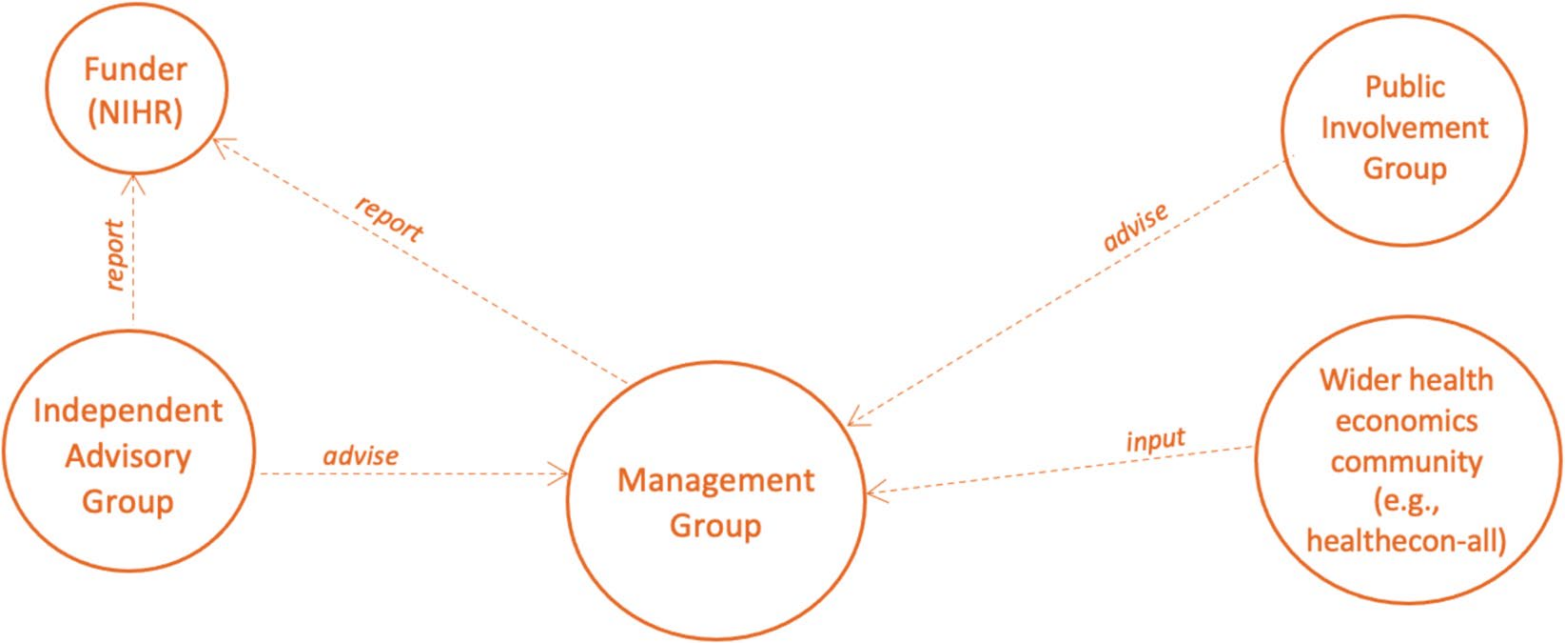
The PRISMA-EconEval project management structure

A core international multidisciplinary working group has been established to oversee all project activities. The PRISMA-EconEval project management structure consists of the PRISMA-EconEval Management Group (i.e. core group of co-applicants of the grant application, the research fellow, and additional expertise) and the PRISMA-EconEval Advisory Group (i.e. a health economist, an information specialist, a systematic reviewer/global health expert, a journal editor, an end-user of health economic evaluations, and a representative of the public—all with independence of the proposed study) (Fig. 1). A separate Public Involvement Reference Group will meet periodically and provide input into the study. We will also solicit input from the wider health economics community at various stages. This study has been registered on the *EQUATOR Network* [20] and the protocol has been published on the *Open Science Framework* [21]. The protocol development adheres to recommended principles for the development of research reporting guidelines [22] and is based on other similar efforts [8–15].

**Fig. 2:**
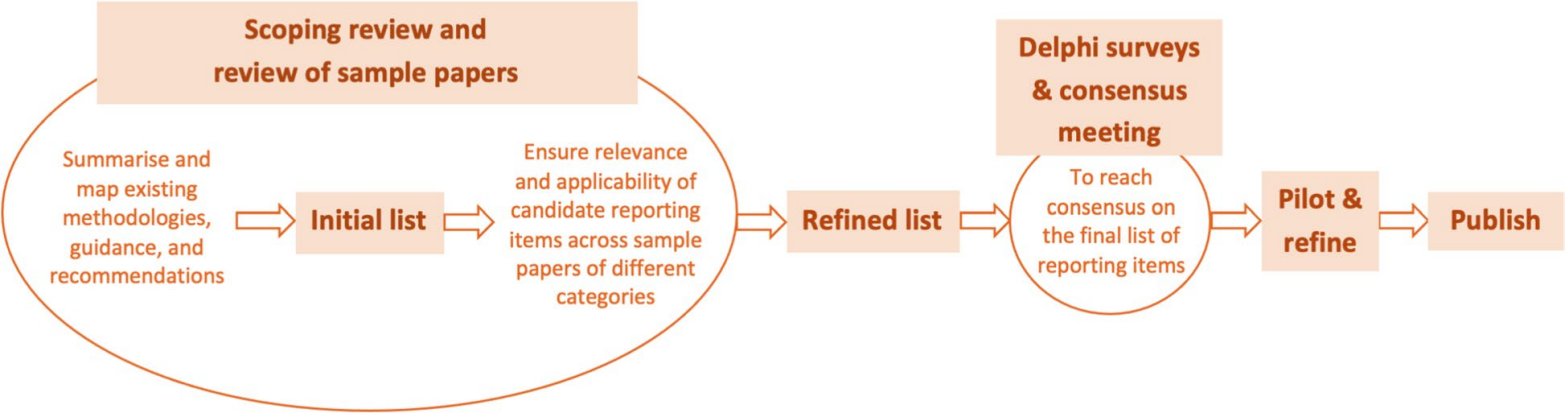
Study process

The study will be conducted in five main stages (Fig. 2). The first will involve the review of the methodological literature on reporting systematic reviews of health economic evaluations (scoping review) and the review of sample systematic reviews of health economic evaluations (review of sample papers). These reviews will be complemented with other pre-Delphi survey activities (e.g. draw upon relevant reporting guidelines such as CHEERS 2022 [17], PRISMA 2020 [2], PRISMA extensions [8, 12, 13], for reference) to generate an initial and then a refined list of reporting items. The second and third stages will consist of a multi-round online Delphi survey and consensus meeting, respectively; and the last two stages will focus on post-consensus meeting activities, including pilot use and dissemination.

### Stage 1: Scoping review and review of samples of systematic reviews of health economic evaluations

The scoping review is the first of a two-part review process. The detailed scoping review protocol has been registered on the *Open Science Framework* [21] and published in F1000Research [23]. The objectives of the scoping review are to identify and review methodological literature, summarise guidance and recommendations, and extract an initial list of candidate reporting items.

The review of sample papers constitutes the second of the two-part review process, aimed at identifying and refining candidate reporting items. The review of sample papers will aim to confirm the global applicability of the candidate items (in view of the important differences in feasibility, local guidance, development, and use of economic evaluations in different countries [24, 25]) and to ensure that they are sufficiently broad to accommodate a set of published systematic reviews, purposively sampled across different review categories.

#### Search strategy and inclusion criteria for the review of samples of systematic reviews

Papers prioritised for inclusion will be systematic reviews of health economic evaluations, purposively sampled from diverse review categories identified in the scoping review. Where necessary, these will be supplemented by community nominations (with the nominating community drawn from sources such as: ISPOR, IHEA, the ‘healthecon-all’ electronic mailing list, comprising approximately 2,500 international members with diverse backgrounds in health economics research, and other sources).

The categories of systematic reviews will be discussed during the PRISMA-EconEval Management Group meetings and likely encompass intervention types such as public health, clinical, and health service delivery [26]. Ensuring that the refined list of candidate reporting items aligns with the sample reviews across categories will enhance the applicability of the final guidelines to all types and purposes of systematic reviews of health economic evaluations.

#### Article screening and selection for the review of samples of systematic reviews

The scoping review team (ABo, ABa, JK, SP, PBT, and ET) will discuss and collectively select two sample papers for each of the subcategories, starting with the sample papers from the scoping review and then extending the search elsewhere, if necessary.

#### Application of the initial list of candidate reporting items on sample systematic reviews

The scoping review team will apply the initial list of candidate reporting items to the selected sample papers and assess the following: [1] Whether the reporting items broadly apply to papers across all categories, [2] whether there are any key reporting items reported in the sample papers that do not appear on our list, and [3] whether the initial list facilitates complete and transparent reporting and supports the quality assessment of both the review and the included studies. A checklist will be developed to facilitate this process and adjustments to the initial list will be made accordingly.

The refined list of candidate reporting items will be used in the subsequent stage of the project (i.e. the Delphi surveys). Each reporting item will be briefly summarised in a statement, accompanied by at least one example of how the item should appear in a systematic review of health economic evaluations. Examples will be drawn from studies identified from previous review processes.

### Stage 2: Multi-round Delphi survey

An online multi-round Delphi survey will be conducted to achieve consensus on items for inclusion in PRISMA-EconEval. The Delphi method, a widely used social scientific approach in healthcare research, organises expert communication around complex issues to achieve consensus non-confrontationally. It has been used previously in the development of health research reporting guidelines [8–17, 27]. It is the recommended approach within published guidance for health research reporting guideline developers to achieve consensus on their content [22]. The Delphi process is informed by the guidance on conducting and reporting Delphi studies (Guidance on Conducting and REporting DElphi Studies — CREDES) [28].

#### Sampling and recruitment of Delphi survey participants

We will follow an inclusive approach to generating a purposive sample for the Delphi surveys. The participant group will comprise academic researchers and other stakeholders, specifically (i) health economists, (ii) systematic reviewers and information specialists, (iii) reporting guideline developers, (iv) journal editors, (v) health care decision-makers (including representatives from local authorities) and health research funders, and (vi) patient and public representatives.

We aim to involve at least 40 participants in each of the groups (i)–(iii) and at least 25 participants in each of the remaining groups, totalling nearly 200 participants in each Delphi rounds, ensuring representation from the World Bank’s low- and middle-income country (LMIC) income groups. To account for an estimated 30% attrition rate, we will initially recruit more participants. Throughout all rounds, participants will receive reminders to complete the surveys and we will extend the survey period if necessary, to minimise attrition and enhance the response rate.

In addition to the identification of potential participants by members of the core PRISMA-EconEval Management Group, we will invite participation from across diverse platforms. These will include targeted mailing lists of professional groups (such as ISPOR, IHEA, the ‘healthecon-all’ electronic mailing list used widely by health economists internationally), social media (e.g. X (formerly Twitter), LinkedIn), and via the EQUATOR Network. We will strive to ensure that the recruitment processes align with the commitments of our host institutions to equality, diversity, and inclusiveness, while also demonstrating international reach. Participation will be confirmed once the invitee acknowledges the participant information sheet and provides written informed consent.

To minimise risk of bias in the Delphi process, responses between rounds will be anonymised so individual judgements remain confidential and uninfluenced. The research team will also use standardised, neutral wording in all communications and avoid one-on-one discussions about Delphi content with participants.

#### Data collection, management, and analysis

A modified Delphi process [29] will be coordinated by the research fellow (PBT) with support from the PRISMA-EconEval Management Group. Panellists will receive a personalised link to a web-based survey, as piloted by the management group members. Informed consent will be obtained from all panellists. The surveys will be conducted using https://app.onlinesurveys.jisc.ac.uk and compliant with General Data Protection Regulation (GDPR). Throughout each round, panellists will remain anonymous to one another and will only be identifiable to the coordinating research fellow. Panellists will not have access to specific answers provided by other members.

##### Round 1

Upon access to the web-based survey, panellists will be asked to consider the following guiding principles when reviewing the initial reporting items generated by the PRISMA-EconEval Management Group: (i) reporting of each item should facilitate complete and transparent reporting of systematic reviews of health economic evaluations, (ii) reporting of each item should facilitate assessment of the quality and applicability of the study findings, (iii) the items should be broadly relevant to all systematic reviews of health economic evaluations, and (iv) the overall set of items should represent the minimum reported in all systematic reviews of health economic evaluations.

The Delphi panellists will then be invited to rate the importance of each of the candidate reporting items identified by the PRISMA-EconEval Management Group on a 9-point Likert scale (1—‘not important’ to 9—‘extremely important’). Each candidate reporting item will include a reporting item description, an associated definition, and a rationale for inclusion. The Delphi panellists will also be invited to describe their confidence in their ratings (‘not confident’, ‘somewhat confident’, or ‘very confident’), comment on the candidate items and their explanations, suggest additional items for consideration in subsequent rounds, and provide any other general comments. Each Delphi panellist will also be asked to provide their gender, region of work, primary and additional work environments, and years of experience. Data will be sent via a secure socket layer (SSL) to a firewalled structured query language (SQL) server at the University of Oxford. Once a round has closed, data will be exported in comma-separated values (CSV) format, and quantitative data will be imported into Stata (version 18; StataCorp, College Station, TX, USA) [30] for analysis.

For each candidate reporting item, measures of central tendency and variability (means and medians, with 95% confidence intervals and interquartile range distributions, as well as a maximum-minimum range for each response, weighted by participant confidence) will be calculated. Items will be removed if > 50% of participants rated an item 3 or lower and < 15% rated it 7 or higher [31] (Table 1). A summary of the ratings and feedback on each remaining item will be created.

**Table 1:** Definition of consensus for reporting items in the Delphi surveys

Round	Consensus	Elaboration	Condition
1	Remove	Consensus that item should not be included in the list	> 50% of participants rated the item ≤ 3 AND < 15% rated it ≥ 7
	Possible	Consensus that item should move to next round	Anything else
2	Include	Consensus that item should be included in the final list	Items with a mean score ≥ 7
	Possible	Consensus that item should be voted on again in a third round	Items with a mean score > 4 and < 7
	Remove	Consensus that item should not be included in the list	Anything else
3 (only for ‘Possible’ items from Round 2)	Include	Consensus that item should be included in the final list	Items with a mean score ≥ 7
	Remove	Consensus that item should not be included in the list	Anything else

##### Round 2

In the second round of the Delphi survey, candidate reporting items will be listed by importance, using the mean score and median score, the inter-percentile range (IPR) (30th and 70th), and the IPR adjusted for symmetry (IPRAS), for each item being rated [32]. Upon viewing these scores, the respondents will be asked to revisit their answers and revise if appropriate. They will be asked to provide a rationale if revising their original estimate and to include evidence that they think is relevant. Participants will be informed that items with a mean score ≥ 7 will be grouped as ‘Included’ reporting items. Items with a mean score > 4 and < 7 will be grouped as ‘Possible’ reporting items and might be excluded from the final checklist. Reporting items with a mean score ≤ 4 will be grouped as ‘Rejected’. Newly introduced items will also be voted on in the second round of the Delphi survey.

##### Round 3

In the event that reporting items are categorised as ‘Possible’ following analyses of round two data, a third round of the Delphi survey will be initiated. In the third round, candidate reporting items will be listed in order of descending importance using the scores from previous rounds along with reasons for high and low importance from previous rounds. The same scoring rules applied in round 2 would be applied in round 3 of the Delphi survey. Delphi panellists will be able to comment on which arguments they found unconvincing and why.

In each round, respondents will be given 14 days to provide responses. Reminders will be sent out seven days and three days before each deadline. Following each round, a week will be allowed for analysis of responses and to accumulate late responses. A flowchart documenting participation will be maintained, including the number of individuals approached and those subsequently unavailable or non-responsive. Sensitivity analyses will be conducted based on respondents’ self-reported confidence in their responses. Where wide variation in responses is reported, mean values may be carried forward from those most confident in their responses.

### Stage 3: Consensus meeting

A consensus meeting will be held virtually and all members of the PRISMA-EconEval Management Group will be invited to attend. In order to achieve a balance of representation across all stakeholder groups, eligible Delphi survey participants may also be invited to attend. Attendance will be limited to 30 participants to encourage spontaneity and maximise interaction in the discussions [22]. The meeting will be audio- and video-recorded and divided into sessions, with chairing responsibilities rotating among members of the PRISMA-EconEval Management Group. All participants will be sent pre-reading materials ahead of the consensus meeting.

The main objectives of the meeting will be as follows: (i) agree on the precise wording of reporting items for which consensus was reached during the online multi-round Delphi survey and (ii) discuss and vote on reporting items for which consensus was not reached during the Delphi surveys. After discussing the reasons for or against inclusion, participants will vote using the *TurningPoint* software [33] to allow for anonymous responses and the final selection decision will be made on the basis of a simple majority. Outcomes from the consensus meeting will be communicated with all Delphi participants.

### Stage 4: Checklist pilot

Following the consensus meeting, academic health economists based at the University of Oxford and the University of Sheffield, who are independent of the development of PRISMA-EconEval, will apply the finalised checklist to a sample of recent systematic reviews of health economic evaluations to identify any practical challenges with the reporting items and to inform the wording of the reporting guidelines. In addition, we will reach out to our networks and invite potential users and interested parties to review and apply the preliminary reporting checklist in order to assess the clarity of wording and to identify remaining gaps or deficiencies. Finally, formal feedback will be collected through a survey of health economists who participated in the initial solicitation of samples of systematic reviews of health economic evaluations in Stage 1.

### Stage 5: Development of PRISMA-EconEval reporting statement and accompanying explanation and elaboration document

Members of the PRISMA-EconEval Management Group will meet in person to: (i) refine the wording and presentation of the reporting checklist based on insights from the piloting and feedback exercises, (ii) assess the placement of each newly identified reporting item and ensure consistency and harmonisation with the original PRISMA 2020 reporting statement, (iii) agree on the processes for finalising the explanation and elaboration document for the PRISMA-EconEval reporting statement, and (iv) develop plans for dissemination and post-publication activities. The Management Group will then draft the final PRISMA-EconEval reporting statement. We anticipate that the statement will be approximately 2,000 words long and will provide a rationale for the development of the reporting guidelines, a description of each stage of the development process, and the final checklist. The PRISMA-EconEval Management Group will also draft an accompanying explanation and elaboration document. This will illustrate each recommended reporting item with at least one exemplar of good practice from the published literature, alongside a detailed explanation of the recommendation.

## Dissemination, outputs, and anticipated impact

To maximise the impact of our study, we will create a dedicated webpage on the Nuffield Department of Primary Care Health Sciences website, regularly updating it as well as social media pages with project progress, findings, and engaging content such as articles, videos, and blogs. The outcome from each of the project stages will be written up as a manuscript and published in a peer-reviewed journal. The PRISMA-EconEval Reporting Statement, together with its explanation and elaboration document, will be published in a peer-reviewed journal, while a downloadable fillable form for authors and video tutorials will be made available online. We will seek wider journal endorsements, co-publication under a Creative Commons licence, and present the statement at academic conferences.

The Management Group will establish a formal dissemination plan to engage major stakeholders. Dissemination will extend to the EQUATOR website, Cochrane, Joanna Briggs Institute, research funders, health technology assessment agencies, and international organisations, mirroring CHEERS [16] and CHEERS 2022 [17] strategies. A comprehensive report for NIHR and a lay-friendly public guide, including glossaries, will be produced and disseminated through NIHR’s ‘Be Part of Research’ initiative [34] and via social media platforms.

While it will be challenging to fully gauge the impact of our research during the funded study period, we will endeavour to track citations and policy impacts using Scopus, Google Scholar, and PlumX [35]. A follow-up study is planned to assess the statement’s continued influence on reporting quality of systematic reviews of health economic evaluations.

## Ethics consideration

Ethics application for the Delphi surveys has been submitted to the University of Oxford’s Central University Research Ethics Committee (CUREC). Delphi panel members will receive a participant information sheet and provide consent at least two weeks before the first survey and the consensus meeting, with an option to opt out of publication acknowledgements. Participants may withdraw at any stage.

Study data will be managed, curated, and stored in accordance with University of Oxford regulations. Aggregated data and literature review outputs will be shared after the study, but individual ratings and consensus meeting transcripts will remain confidential.

## Project management

The PRISMA-EconEval Management Group meets monthly during the 12-month study period to oversee activities, address challenges, and ensure milestones are met; including protocol development, ethical approval, study material preparation, Delphi survey development and deployment, consensus meeting planning, and piloting. Meetings are mainly held online.

The Advisory Group will meet three times to provide independent advice on the design, conduct, analysis, and dissemination.

## Patient and public involvement (PPI)

Patient and public involvement will be integrated into all stages of the development of the PRISMA-EconEval reporting guidelines. A public contributor group of nine individuals with experience in applied health research has been formed, including members of the CHEERS 2022 Public Involvement Reference Group and additional members from diverse backgrounds.

Five online meetings will guide the group’s involvement, starting with establishing roles and working methods and identifying needs. UK PPI Standards will shape the approach, complemented by bespoke trainings, health economic evaluation discussions, and access to the University of Southampton’s public involvement foundation course.

Public contributors will assist in developing data extraction plans, interpreting study results, identifying candidate reporting items, participating in each Delphi round, and contributing to the final consensus meeting. To ensure meaningful engagement, we will provide key information before each meeting, support thorough preparation, and compensate contributors for their time.

Meetings will be held virtually, with support for technology access as needed. Sophie Staniszewska and Richard Grant will coordinate, with chair/co-chair rotation discussed in the first meeting. Meetings will be recorded and transcribed to document contributions, which will be reported as a main paper using GRIPP2 [36] or as a commentary, as done for CHEERS 2022 [37].

## Discussion

We aim to provide a further extension to PRISMA 2020 for the reporting of systematic reviews of health economic evaluations (PRISMA-EconEval). The development of PRISMA-EconEval will enhance completeness, transparency, and structure in the reporting of systematic reviews of health economic evaluations and generate user-friendly tools that facilitate reporting for authors, editors, peer-reviewers, and stakeholders.

A significant strength of the study will be its inclusive review of both methodological papers and samples of systematic reviews, along with the involvement of diverse stakeholders and the broader scientific and lay community. This inclusive approach will ensure that the perspectives of users and beneficiaries are considered. By aiming for approximately 200 global participants in the Delphi process, the study will generate items and achieve consensus from various contexts and needs.

The research team includes experienced researchers and experts in evidence synthesis, health economics, and methodology and guideline development. The support from PRISMA, the Patient and Public Involvement and Engagement network, and the extensive network of 2,500 health economists (‘healthecon-all’) will help identify appropriate stakeholders for the community nomination of sample papers, the Delphi panel, the consensus process, and the dissemination of the study’s outcomes. Success criteria will be measured according to our milestones and timelines.

We anticipate two main challenges to successful completion of the study. First is the challenge posed by identification of relevant systematic reviews of health economic evaluations through searches of the published and grey literature and contact with health economists to inform a comprehensive initial list of reporting items for evaluation. The co-principal applicants have published several systematic reviews of health economic evaluations. Furthermore, our information specialist co-applicant has substantial experience in information retrieval and has authored numerous articles and book chapters in this field. He will advise on overall search strategies (e.g. sources, limits) and on the selection of search terms and syntax. He will design a strategy that optimises sensitivity and specificity for both the methodological literature and sample papers.

Second is the sufficient and timely recruitment and retention of a broad set of stakeholders for the online multi-round Delphi survey and the subsequent consensus meeting. Members of the PRISMA-EconEval Management Group have previously recruited diverse sets of stakeholder groups for several reporting guidelines initiatives (e.g. CONSORT, SPIRIT, CHEERS, CHEERS 2022, MAPS, RETRIEVE, GRIPP2). We will build on this experience, with a particular focus on lessons learned about recruiting from underrepresented groups; e.g. patient and public representatives, health technology assessment agency representatives, and other end-users of health economic evaluations.

Future research planned by the PRISMA-EconEval Management Group includes a before-and-after evaluation of the benefits (and indeed, possible adverse effects) of the introduction of the PRISMA-EconEval reporting statement. It will also be necessary to update the PRISMA-EconEval reporting statement in the future to address conceptual, methodological, and practical advances in the field.

## Data Availability

Not applicable.

## Abbreviations

CC BY: Creative Commons license
CHEERS: Consolidated Health Economic Evaluation Reporting Standards
CONSORT: Consolidated Standards of Reporting Trials
CREDES: Guidance on Conducting and REporting DElphi Studies
CSV: Comma-separated values
CUREC: University of Oxford’s Central University Research Ethics Committee
EQUATOR: Enhancing the quality and transparency of health research
GDPR: General Data Protection Regulation
GRIPP2: Guidance for Reporting Involvement of Patients and the Public 2
IPR: Inter-percentile range
IPRAS: Inter-percentile range adjusted for symmetry
LMIC: Low- and middle-income country
MAPS: Preferred Reporting Items for Studies Mapping onto Preference-Based Outcome Measures
NIHR: National Institute for Health and Care Research
PPI(E): Patient and public involvement (and engagement)
PRISMA-EconEval: PRISMA Extension for Systematic reviews of Health Economic Evaluations
PRISMA-ScR: PRISMA Extension for Scoping Reviews
PRISMA: Preferred Reporting Items for Systematic reviews and Meta-Analyses
RETRIEVE: Checklist for Studies Reporting the Elicitation of Stated Preferences for Child Health-Related Quality of Life
SOPs: Standard operating procedures
SPIRIT: Standard Protocol Items: Recommendations for Interventional Trials
SQL: Structured query language
SSL: Secure socket layer
